# Sex-specific impact of prenatal androgens on social brain default mode subsystems

**DOI:** 10.1038/s41380-018-0198-y

**Published:** 2018-08-13

**Authors:** Michael V. Lombardo, Bonnie Auyeung, Tiziano Pramparo, Angélique Quartier, Jérémie Courraud, Rosemary J. Holt, Jack Waldman, Amber N. V. Ruigrok, Natasha Mooney, Richard A. I. Bethlehem, Meng-Chuan Lai, Prantik Kundu, Edward T. Bullmore, Jean-Louis Mandel, Amélie Piton, Simon Baron-Cohen

**Affiliations:** 1 Center for Applied Neuroscience, Department of Psychology, University of Cyprus, Nicosia, Cyprus; 2 Autism Research Centre, Department of Psychiatry, University of Cambridge, Cambridge, United Kingdom; 3 Department of Psychology, School of Philosophy, Psychology, and Language Sciences, University of Edinburgh, Edinburgh, United Kingdom; 4 Department of Neurosciences, University of California, San Diego, CA USA; 5 Institut de Génétique et de Biologie Moléculaire et Cellulaire, Illkirch, France; 6 Centre National de la Recherche Scientifique, UMR7104 Illkirch, France; 7 Institut National de la Santé et de la Recherche Médicale, U964 Illkirch, France; 8 Université de Strasbourg, Illkirch, France; 9 Child and Youth Mental Health Collaborative, Centre for Addiction and Mental Health and the Hospital for Sick Children, Department of Psychiatry, University of Toronto, Toronto, ON Canada; 10 Department of Psychiatry, National Taiwan University Hospital and College of Medicine, Taipei, Taiwan; 11 Section on Advanced Functional Neuroimaging, Departments of Radiology & Psychiatry, Icahn School of Medicine at Mount Sinai, New York, NY USA; 12 Brain Mapping Unit, Department of Psychiatry, University of Cambridge, Cambridge, United Kingdom; 13 Cambridgeshire and Peterborough National Health Service Foundation Trust, Cambridge, United Kingdom; 14 ImmunoPsychiatry, GlaxoSmithKline Research and Development, Stevenage, United Kingdom; 15 Chair of Human Genetics, Collège de France, Paris, France

**Keywords:** Autism spectrum disorders, Neuroscience, Genetics, Psychiatric disorders

## Abstract

Early-onset neurodevelopmental conditions (e.g., autism) affect males more frequently than females. Androgens may play a role in this male-bias by sex-differentially impacting early prenatal brain development, particularly neural circuits that later develop specialized roles in social cognition. Here, we find that increasing prenatal testosterone in humans is associated with later reduction of functional connectivity between social brain default mode (DMN) subsystems in adolescent males, but has no effect in females. Since testosterone can work directly via the androgen receptor (AR) or indirectly via the estrogen receptor through aromatase conversion to estradiol, we further examined how a potent non-aromatizable androgen, dihydrotestosterone (DHT), acts via the AR to influence gene expression in human neural stem cells (hNSC)—particularly for genes of high-relevance for DMN circuitry. DHT dysregulates a number of genes enriched for syndromic causes of autism and intellectual disability and for genes that in later development are expressed in anatomical patterns that highly correspond to the cortical midline DMN subsystem. DMN-related and DHT-affected genes (e.g., *MEF2C*) are involved in a number of synaptic processes, many of which impact excitation-inhibition balance. Androgens have male-specific prenatal influence over social brain circuitry in humans and may be relevant towards explaining some component of male-bias in early-onset neurodevelopmental conditions.

## Introduction

It has long been known that events occurring during prenatal development can have long-lasting programming impact on susceptibility for medical conditions that emerge later in life [1–3]. Emerging work has shown that events starting during prenatal development can have long-term impact potentially leading to increased likelihood for atypical neurodevelopmental phenotypes. For instance, neurodevelopmental conditions such as autism have been linked to multiple types of biological processes that stem from very early periods of prenatal development [4–10]. Therefore, it is becoming increasingly clear that prenatal brain development is a critical window of importance for understanding factors that increase the likelihood of neurodevelopmental conditions.

Several early-onset neurodevelopmental conditions (e.g., autism, intellectual disability, attention deficit hyperactivity disorder (ADHD), developmental language disorders, conduct disorder) are well known to have a sex-biased prevalence rate [11]. For example, the latest estimates for autism suggest that three males are diagnosed for every one female [12]. Several theories have been put forward to explain the sex ratio imbalance in early-onset neurodevelopmental conditions [13], over and above under-diagnosis or mis-diagnosis in females [14]. One prominent theory suggests that there are factors inherent in females that act to reduce the likelihood of atypical neurodevelopment [15]. For example, a higher burden of large effect mutations are present in females compared to males with autism, suggesting that protective mechanisms in females may raise the threshold for deleterious impact of such mutations [16]. In contrast to female-specific factors reducing likelihood of autism, there may also be important male-specific mechanisms for increasing the likelihood of developing autism. We have theorized that sex steroid hormones such as testosterone may have fetal programming effects on later brain development in ways relevant to autism [17]. Recent large studies have confirmed that maternal polycystic ovary syndrome, a syndrome associated with elevated androgen levels, increases the odds for both autism and ADHD in offspring [18–20]. We also recently confirmed elevation of a latent steroidogenic factor affecting multiple steroid hormones including testosterone in the amniotic fluid of clinically diagnosed males with autism [7]. Extending into the non-clinical population, continuous variation in how much fetal testosterone (FT) an individual is exposed to in the womb is associated with behavioral variation in autistic traits [21, 22]. FT and later postnatal testosterone variation also affects specific behavioral and cognitive domains such as social cognition, language, emotion, and reward processes, which are implicated in autism and other male-biased neurodevelopmental conditions [23–25]. Thus, it may be that multiple sex-specific factors could be at work to both increase likelihood in males and decrease likelihood in females for developing early-onset neurodevelopmental conditions. Further work is needed to tease apart how these mechanisms may operate similarly or differently in males and females. In addition, translational work is needed to examine how such mechanisms directly affect biological processes supporting both typical and atypical human brain development.

In this study, we first examine the question of whether FT longitudinally exerts sex-specific influence over intrinsic functional organization of specific circuits involved in social, language, and affective functions in humans. To test this question, we examined a unique cohort of individuals in whom we have measured concentration levels of testosterone directly from amniotic fluid during a midgestational window of pregnancy where fetuses are sexually differentiating and where surges in sex steroid levels may have maximal impact on early brain development [26, 27]. These individuals are now in their adolescent years and we examined how variability in FT is associated with patterns of intrinsic functional connectivity as measured with resting state functional magnetic resonance imaging (rsfMRI). Given known links between FT and the behavioral and cognitive domains of social cognition, social-communication, language, emotion, and reward [23, 24], we examined specific neural circuits known for their roles in these domains. We predicted that FT would act as a male-specific mechanism to influence connectivity towards atypicality and that such male-specific influence would not be observed in females.

While it is important to identify associations between FT and macroscale neural circuitry, we also want to better understand how androgens like testosterone exert mechanistic influence over early prenatal neural development. Testosterone can exert gene regulatory influence through several routes. Testosterone or conversion via 5-alpha-reductase to dihydrotestosterone (DHT) could potentially act in a direct manner via the androgen receptor (AR) to influence transcription of other genes targeted by the AR. However, testosterone can also be converted to estradiol via aromatase and have different transcriptional influence over genes targeted by the estrogen receptor (ER). To understand how testosterone may have AR-dependent influence on early neural development we further examined how DHT affects gene expression in human neural stem cells (hNSC) [28]. Of particular interest for this work is the overlap between DHT-dysregulated genes and genes spatially expressed in similar patterns across cortex as rsfMRI-defined macroscale networks that are associated with FT. If FT influences later neural circuitry in an AR-dependent manner, we expect that the DHT-dysregulated gene set would be enriched for genes that are spatially expressed in patterns that highly resemble FT-associated rsfMRI networks.

## Methods

### Participants

This study was approved by the Essex 1 National Research Ethics committee. Parents gave informed consent for their child to participate and each adolescent also gave assent to participate. Participants were 64 adolescents (32 males, 32 females; male age mean = 15.42 years, standard deviation = 0.92 years; female age mean = 15.55 years, standard deviation = 1.06 years; age range = 13.22–17.18 years) sampled from a larger cohort of individuals whose mothers underwent amniocentesis during pregnancy for clinical reasons (i.e., screening for chromosomal abnormalities). At amniocentesis, none of the individuals screened positive for any chromosomal abnormalities and were thus considered typically developing. At the time of scanning, none of the participants self- or parent-reported any kind of neurological or psychiatric diagnosis. After assays of current testosterone levels were completed, we found that the assay did not result in useable data for four males and two females, and thus these individuals were excluded from further analyses requiring intact FT and current testosterone data.

### Fetal testosterone collection and measurement

FT was measured from amniotic fluid samples collected between 13 and 20 weeks of gestation via radioimmunoassay. This period is within the 8–24 week window that is hypothesized to be critical for human sexual differentiation [26]. Amniotic fluid was extracted with diethyl ether, which was evaporated to dryness at room temperature and the extracted material redissolved in an assay buffer. Testosterone was assayed by the Count-a-Coat method (Diagnostic Product), which uses an antibody to testosterone coated onto propylene tubes and a 125I-labeled testosterone analog. The detection limit of the assay using the ether-extraction method is 0.05 nmol/L. The coefficient of variation (CV) for between-batch imprecision is 19% at a concentration of 0.8 nmol/L and 9.5% at a concentration of 7.3 nmol/L. The CVs for within- batch imprecision are 15% at a concentration of 0.3 nmol/L and 5.9% at a concentration of 2.5 nmol/L. This method measures total extractable testosterone.

### Current testosterone collection and measurement

Current testosterone during adolescence was measured from passive saliva samples using a commercial competitive ELISA (Salimetrics Ltd, US) at the Biomarker Analysis Laboratory at Anglia Ruskin University. All pipetting used a Tecan Evo liquid handler. Twenty-five microliters of saliva samples, standard and controls were pipetted into the appropriate wells of the ELISA plates, 150 µL of testosterone conjugated to horseradish peroxidase (HRP) enzymes was added to all wells and the plate shaken for 5 min and then incubated for 55 min at room temperature. The ELISA plates were washed four times in Salimetrics wash buffer using a Tecan Hydroflex before the addition of 200 µL of enzyme substrate (TMB), the plates shaken for 5 min and color allowed to develop for an additional 25 min before the reaction was stopped by the addition of 50 µL of Salimetrics stop solution. The resulting optical density in the wells was read at 450 nm with a secondary filter correction at 620 nm. The sensitivity of this assay is 1 pg/mL. The CVs for within- batch imprecision were 4.53% for the high controls and 14.88% for the low controls.

### fMRI data acquisition

All MRI scanning took place on a 3T Siemens Tim Trio MRI scanner at the Wolfson Brain Imaging Center in Cambridge, UK. Functional imaging data were acquired with a multi-echo echo planar imaging (EPI) sequence with online reconstruction (repetition time (TR), 2000 ms; field of view (FOV), 240 mm; 28 oblique slices, descending alternating slice acquisition, slice thickness 3.8 mm; echo times (TE) = 13, 31, and 48 ms, GRAPPA acceleration factor 2, BW = 2368 Hz/pixel, flip angle, 90°, voxel size 3.8 mm isotropic). Resting state data were collected using a 10 min ‘‘eyes-open’’ run (i.e., 300 volumes), where participants were asked to stare at a central fixation cross and to not fall asleep. Anatomical images were acquired using a T1-weighted magnetization prepared rapid gradient echo (MPRAGE) sequence for warping purposes (TR, 2300 ms; TI, 900 ms; TE, 2.98 ms; flip angle, 9°, matrix 256 × 256 × 256, field-of-view 25.6 cm).

### fMRI preprocessing

Data were processed by ME-ICA using the tool meica.py as distributed in the AFNI neuroimaging suite (v2.5), which implemented both basic fMRI image preprocessing and decomposition-based denoising. For the processing of each subject, first the anatomical image was skull-stripped and then warped nonlinearly to the MNI anatomical template using AFNI *3dQWarp*. The warp field was saved for later application to functional data. For each functional dataset, the first TE dataset was used to compute parameters of motion correction and anatomical-functional coregistration, and the first volume after equilibration was used as the base EPI image. Matrices for de-obliquing and six-parameter rigid body motion correction were computed. Then, 12- parameter affine anatomical-functional coregistration was computed using the local Pearson correlation (LPC) cost function, using the gray matter segment of the EPI base image computed with AFNI *3dSeg* as the LPC weight mask. Matrices for de-obliquing, motion correction, and anatomical-functional coregistration were combined with the standard space non-linear warp field to create a single warp for functional data. The dataset of each TE was then slice-time corrected and spatially aligned through application of the alignment matrix, and the total non-linear warp was applied to the dataset of each TE. No time series filtering was applied in the preprocessing phase. Data were analyzed with no spatial smoothing. ME-ICA denoising was used to identify and remove non-BOLD signal fluctuation [29–31].

### Group independent components analysis and dual regression

To assess large-scale intrinsic functional organization of the brain we utilized the unsupervised data-driven method of independent component analysis (ICA) to conduct a group-ICA, and then utilized dual regression to back-project spatial maps and individual time series for each component and subject. Both group-ICA and dual regression was implemented with FSL’s MELODIC and Dual Regression tools (www.fmrib.ox.ac.uk/fsl). For group-ICA, we constrained the dimensionality estimate to 30, as in most cases with low-dimensional ICA, the number of meaningful components can be anywhere from 10–30 [32]. From these components, we manually selected the components that best represented networks typically involved in emotion (amygdala), reward (ventral striatum), language (superior temporal gyrus, inferior frontal gyrus, insula), and social cognition (default mode network) functions.

### Between-component connectivity analysis

Time courses for each subject and each component were used to model between-component connectivity. This was achieved by constructing a partial correlation matrix for all six components using Tikhonov-regularization (i.e., ridge regression, rho = 1) as implemented within the *nets_netmats.m* function in the FSLNets MATLAB toolbox (https://fsl.fmrib.ox.ac.uk/fsl/fslwiki/FSLNets). The aim of utilizing partial correlations was to estimate direct connection strengths in a more accurate manner than can be achieved with full correlations, which allow more for indirect connections to influence connectivity strength [32–34]. Partial correlations were then converted into Z-statistics using Fisher’s transformation for further statistical analyses. The lower diagonal of each subject’s partial correlation matrix was extracted for a total of 15 separate component-pair comparisons. For each of these 15 comparisons we ran robust regression (to be insensitive to outliers) [35] (https://github.com/canlab/RobustToolbox) to examine correlations between connectivity and FT variation. This analysis was conducted twice—once with FT only and again with FT and controlling for variability in current levels of testosterone. These correlations were computed separately for males and females. Finally, we computed Z-statistics and *p*-values for difference between male and female correlations utilizing the *paired.r* function in the *psych* R library. False positive control was achieved with FDR *q* < 0.05, implemented with the *p.adjust* function in R.

### Androgen influence on gene expression in a human neural stem cell model

To gain insight into the impact of androgens on gene expression in embryonic development, we examined data from a recent RNA-seq experiment whereby human neural stem cells (hNSC) derived from embryonic stem cells were treated with 100 nM of a potent non-aromatizable androgen, dihydrotestosterone (DHT) or a control treatment (dimethyl sulfoxide; DMSO) [28] (GEO accession number: GSE86457). The cell line used was line SA001 from a male donor, obtained from Cellartis (Goteborg, Sweden). The work was supervised by the French Bioethics Agency (Agreement number NOR AFSB 12002 43S). hNSCs were derived as described in Boissart et al. [36]. Read counts obtained from RNA sequencing of three batches (biological replicates) of treated hNSCs derived from SA001 (DMSO (*n* = 4;3;3) vs. DHT 100 nM (*n* = 3;3;3)) were analyzed, scaling them by library size using the trimmed mean of M values (TMM) method, implemented with the calcNormFactors function in the edgeR library [37]. Low expressing genes were removed if there was not two or more samples with more than 100 reads while the previous analysis [28] filtered out genes with less than 100 reads normalized and divided by gene length in kb for each condition and below the 80th percentile in one of the condition studied (DMSO or DHT 100 nM 24 h). This filtering left a total of 13,284 genes for further downstream analysis. Batch effects were removed using the *ComBat* function within the *sva* R library. The software utilized in the prior paper [28] for DE analysis was DESeq2 [38]. This study used the *voom* function from the *limma* library in R to estimate precision weights for linear modeling of differential expression (DE) that will account for mean-variance trends [39]. DE analysis in *limma* allows for utilization of the *voom* function to estimate precision weights that account for mean-variance trends and which are incorporated directly into linear DE models. Another unique aspect of DE analysis within *limma* is the incorporation of an empirical Bayes procedure for sharing information between genes in estimating variance. Genes were identified as DE if they survived Storey FDR correction at *q* < 0.05 [40].

### Isolating DMN-relevant genes based on spatial expression similarity to rsfMRI components

We next wanted to isolate genes with high-relevance to our specific large-scale neural circuits identified by rsfMRI ICA analysis. To achieve this aim, we used the gene expression decoding functionality within Neurosynth and NeuroVault [41] to identify genes whose spatial expression patterns are consistently similar across subjects to our rsfMRI IC maps. This decoding analysis utilizes the six donor brains from the Allen Institute Human Brain Gene Expression atlas [42, 43]. The analysis first utilizes a linear model to compute similarity between the observed rsfMRI IC map and spatial patterns of gene expression for each of the six brains in the Allen Institute dataset. The slopes of these subject-specific linear models encode how similar each gene’s spatial expression pattern is with our rsfMRI IC maps. These slopes were then subjected to a one-sample *t*-test to identify genes whose spatial expression patterns are consistently of high similarity across the donor brains to the rsfMRI IC maps we input. The resulting list of genes was then thresholded for multiple comparisons and only the genes surviving FDR *q* < 0.05 and with positive *t*-statistic values were considered.

### Gene set enrichment analyses

Analyses examining enrichment (i.e., overlap) between two lists of genes, was implemented using the *sum(dhyper)* function in R. For enrichments with Neurosynth Gene Expression Decoding analyses, the background set size for all enrichment analyses was set to the total number of genes considered for Neurosynth Gene Expression Decoding analyses (i.e., 20,787). We also examined enrichment with autism-associated genes listed under SFARI Gene Scoring categories (lists downloaded on 10/10/2017). The background set size for this analysis was set to the total number of genes included in the DE analysis (13,284).

All enrichment analyses using Gene Ontology (GO) database was implemented with AmiGO 2 (http://amigo.geneontology.org/amigo). Here we used a custom background list, which was the total number of genes included in the DE analysis (i.e., 13,284 genes). Only GO terms surviving Bonferroni correction were used.

### Developmental trajectory of MEF2C expression

RNA-seq data from the Allen Institute BrainSpan Human Brain Gene Expression atlas was utilized for this analysis. We examined medial prefrontal cortex (MFC) in BrainSpan as it is the most prominent region from the IC01 functional connectivity map (see Results). We tested hypotheses about whether there is developmental upregulation of *MEF2C* and enhanced variability in prenatal vs. postnatal development. To test this, we used a permutation test (100,000 permutations) whereby on each permutation we randomized prenatal or postnatal labels and then re-calculated the mean difference or difference in standard deviation in expression between prenatal and postnatal (after birth) periods. We then compared the observed mean or standard deviation difference statistics to their null distributions to compute the *p*-value for each comparison.

### Follow-up experiment of DHT-dysregulation of MEF2C expression using qPCR

Three lines of hNSCs cells (two derived from iPSC lines reprogrammed from male fibroblasts GM01869 and GM04603 and one derived from blood of an anonymous female donor, PB12) were treated by DMSO or DHT 100 nM during 24 h, in quadruplicates, as previously described in Quartier et al. [28]. RNA was extracted using RNeasy minikit (Qiagen, Valencia, CA, USA) including a DNase I treatment. Total RNA (500 ng) was reverse transcribed into cDNA using random hexamers and SuperScript II reverse transcriptase according to the manufacturer’s recommendation (Invitrogen, Carlsbad, CA, USA). Real-time PCR quantification (qPCR) was performed on cDNA on LightCycler 480 II (Roche) using the QuantiTect SYBR Green PCR Master Mix (Qiagen) and primers specific to *MEF2C* (MEF2C_RT_F: 5′-ATCGACCTCCAAGTGCAGGTAACA-3′ and MEF2C_RT_R: 5′- AGACCTGGTGAGTTTCGGGGATT-3′). All qPCR reactions were performed in triplicate. Reaction specificity was controlled by post-amplification melting curve analysis. The relative expression of gene-of-interest vs. two references genes (*GAPDH* and *YWHAZ*) was calculated using the 2-(ΔΔCt) method. To test for DHT upregulation of *MEF2C* expression we utilized a linear mixed-effect model (*lme* function within the *nlme* R library) with condition and sex as fixed effects and cell line and replicate as crossed random effects. To quantify evidence of replication of the original *MEF2C* result in RNA-seq data, we computed a replication Bayes Factor [44]. Replication Bayes Factors >10 indicate strong evidence for replication.

### MEF2C expression in an iPSC model of autism

*MEF2C* expression was examined from the RNA-seq data from Marchetto et al. [10]. This dataset comprises expression measured from induced pluripotent stem cells (iPSC), neural progenitor cells, and neurons grown from fibroblasts of typically developing controls or patients with autism. Analyses were specifically focused on *MEF2C* expression and used a linear mixed-effect model ANOVA (*lme* function from the *nlme* R library) modeling diagnosis, RIN, cell type, and diagnosis⁎cell type interaction as fixed effects and subject identifier as a random effect. This ANOVA was followed-up by specific tests (*lm* function in R) of between-group difference within each cell type, covarying for RIN.

### Code availability

The code used to reproduce the findings of this study are available from the corresponding author upon reasonable request.

## Results

### Sex-differential FT influence on connectivity between social brain default mode subsystems

**Fig. 1:**
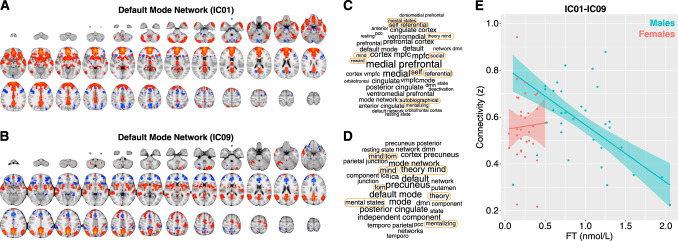
Sex-differential relationship between FT and DMN subsystem connectivity. **a**, **b** show axial montages of the two DMN components (IC01, IC09). **c** Shows a scatterplot of the relationship between FT and IC01-IC09 connectivity

Confirming our overall hypothesis that FT exerts sex-specific influence over connectivity between neural circuits underpinning functions affected in male-biased neurodevelopmental conditions, we find only one between-component connection that is differentially related to FT in males vs. females. This between-component connection comprises anterior and primarily cortical midline (IC01) vs. posterior (IC09) components of the default mode network (DMN) (Fig. 1a, b). On average, connectivity between these components is robustly non-zero indicating strong normative relationships between these two integral parts of the DMN. However, as FT increases, connectivity between IC01 and IC09 decreases, specifically for males but not females (male *r* = −0.69, *p* = 0.0001; female *r* = 0.02, *p* = 0.89; *z* = 3.35, *p* = 7.88e-4) (Fig. 1c). In other words, within males specifically, increasing FT has an effect of reducing connectivity between these DMN subsystems. In contrast to the sex-specific effects identified here, we also analyzed the data ignoring sex as a factor. However, we did not find any evidence of a relationship between FT and DMN connectivity that spans across the sexes (*r* = −0.20, *p* = 0.15; Supplementary Fig. 1). Because all subjects were adolescents, current levels of testosterone could play a potential mediating role in the relationship between FT and DMN connectivity. FT and current testosterone were not correlated in females (*r* = 0.03, *p* = 0.86), but showed a positive trend in males (*r* = 0.33, *p* = 0.08) (Supplementary Fig. 2). Current testosterone was also significantly negatively correlated with DMN connectivity in males (male *r* = −0.37, *p* = 0.008), but not females (female *r* = 0.08, *p* = 0.97) and there was no evidence of difference in the strength of the correlations (*z* = 1.55, *p* = 0.12) (Supplementary Fig. 3). When controlling for the effect of current testosterone, we find that sex-specific relationships between FT and DMN connectivity persist (male *r* = −0.63, *p* = 0.001; female *r* = 0.07, *p* = 0.74; *z* = 3.12, *p* = 0.001), indicating that this effect is not explained by current levels of testosterone during adolescence.

### Genes influenced by androgens and relevant to DMN circuits

We next asked the question of how androgens may impact gene expression in early prenatal development—specifically DMN circuitry. To model these early stages of development, we re-analyzed previously published [28] transcriptomic data obtained from a human neural stem cell model (hNSC) treated with a potent non-aromatizable androgen, dihydrotestosterone (DHT, 100 nM). Differential expression (DE) analysis identified 460 genes upregulated and 221 genes downregulated by DHT. Upregulated genes are enriched in numerous processes spanning angiogenesis, blood vessel morphogenesis and development, enzyme linked receptor protein signaling, cell surface receptor signaling, signal transduction, cell morphogenesis, neuron development, and cell differentiation, among many others. Downregulated genes are enriched in cardiac chamber morphogenesis, regionalization, pattern specification process, neuron differentiation, neurogenesis, among many others (Supplementary Fig. 4A, B). We previously reported enrichments with many autism-associated genes, which are also known as syndromic causes of autism [28]. Although our DE analyses differed from the past paper [28], the current enrichment analyses largely confirm this prior finding. Many genes listed in the ‘‘Syndromic’’ category of SFARI Gene were also DHT-dysregulated (e.g., *HI1*, *ASXL3*, *CHD2*, *DHCR7*, *HCN1*, *MEF2C*, *PAX6*, *PRODH*, *PTEN*, and *SCN1A*). Other important autism-associated genes were also DHT-dysregulated such as *NLGN4X*, *NRXN3*, *FOXP1*, and *SCN9A*. For more on this enrichment analysis between DHT-dysregulated genes and autism-associated genes for this analysis, see the Supplementary Results and Supplementary Fig. 4C.

**Fig. 2:**
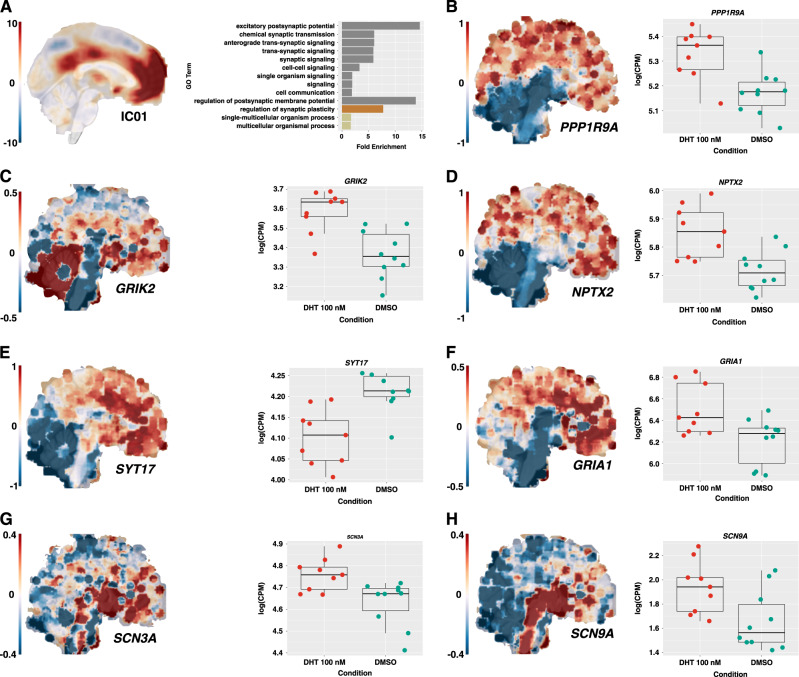
Synaptic enrichments of DHT-dysregulated genes and genes with high levels of spatially expression similarity to rsfMRI DMN IC01 map (**a**) and plots of specific genes contributing to these enrichments (**b**–**h**). Whole-brain maps showing expression for each gene are composite maps averaging across all donors. These composite maps are shown for visualization purposes only. They are not meant to reflect directly the hierarchical statistical testing as implemented with Neurosynth Gene Expression Decoding. The coloring in the enrichment plot in **a** represents terms from different Gene Ontology (GO) clusters

We then tested the critical question regarding whether any genes influenced by DHT in hNSCs are relevant for developing cortical networks such as the DMN, which are influenced by FT in a sex-specific manner. We used Neurosynth Gene Expression Decoding analyses to isolate spatial gene expression patterns that are similar to the DMN components identified by rsfMRI. While only four genes pass at FDR *q* < 0.05 for the IC09 component, 2444 genes pass at the same FDR threshold for the IC01 component. This IC01 gene set was significantly enriched in genes that are differentially expressed by DHT in hNSCs (OR = 1.88, *p* = 0.000002). This overlapping gene set was highly enriched in a number of synaptic processes (Fig. 2a). Several examples of such genes are shown in Fig. 2b–f—particularly myocyte enhancer factor 2C (*MEF2C*), synaptotagmin 17 (*SYT17*), neurabin-1 (*PPP1R9A*), neuronal pentraxin 2 (*NPTX2*), glutamate receptor 1 (*GRIA1*), and glutamate receptor, ionotropic, kainate 2 (*GRIK2*), sodium channel, voltage gated, type III alpha subunit (*SCN3A*), and sodium channel, voltage gated, type IX alpha subunit (*SCN9A*).

### Similar directionality of MEF2C dysregulation in induced pluripotent stem cells of male patients with autism

**Fig. 3:**
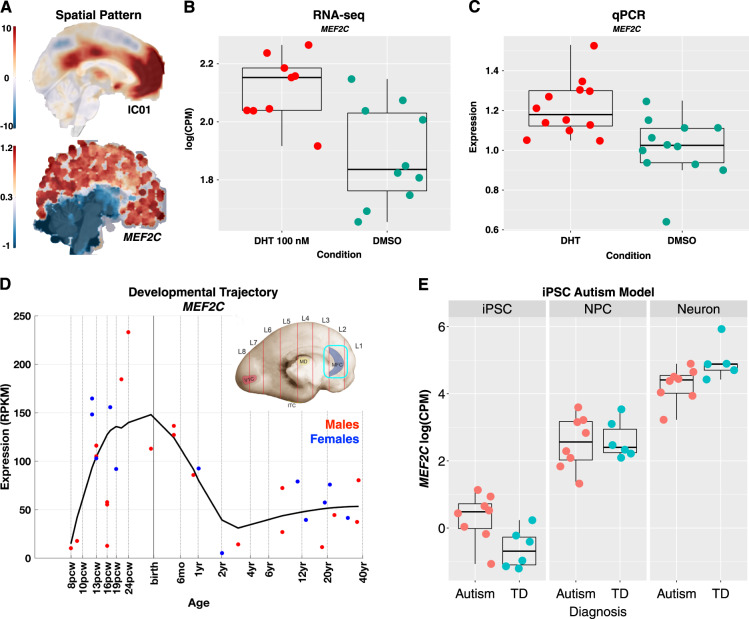
**a** Shows spatial pattern of *MEF2C* expression compared to spatial rsfMRI map for IC01. The whole-brain *MEF2C* expression maps shown is a composite map averaging across all donors. This composite map is shown for visualization purposes only. It is not meant to reflect directly the hierarchical statistical testing as implemented with Neurosynth Gene Expression Decoding. **b**, **c** Show expression of *MEF2C* across RNA-seq (**b**) and qPCR (**c**) experiments. **d** Shows the developmental trajectory of *MEF2C* expression in the Allen Institute BrainSpan atlas (blue, female; red male). **e** Shows *MEF2C* expression across induced pluripotent stem cells (iPSC), neural progenitor cells (NPC), and neurons from cases with autism or typically developing controls (TD)

Of the genes that are both differentially expressed by DHT and spatially highly expressed in a pattern associated with the DMN, we followed-up one gene of particular interest—*MEF2C* (Fig. 3a). *MEF2C* is among the genes from SFARI Gene noted as a syndromic cause of autism and is differentially expressed here by DHT. *MEF2C* is also known to be a downstream target of the androgen receptor [45]. While *MEF2C* itself is not sex-differentially expressed in the brain during prenatal and adult periods of development [46, 47], as a transcription factor it can differentially target other genes as a function of sex and the degree of such sex-differential targeting explains a substantial amount of variance in sex-differential expression [48]. Confirming DHT upregulation of *MEF2C* expression we ran a follow-up experiment using qPCR and found that indeed the upregulation of *MEF2C* by DHT in the RNA-seq data is replicated using qPCR on three additional independent cell lines (*t* *=* 3.73*, p* = 0.003*, replication Bayes Factor* = 164) (Fig. 3b, c).

We went further in subsequent analyses to identify *MEF2C*’s trajectory of expression in medial prefrontal cortex throughout prenatal and postnatal periods. Using the Allen Institute BrainSpan Developmental Gene Expression atlas we indeed find that prenatal development is a prominent period where *MEF2C* is upregulated in medial prefrontal cortex (*p* = 0.01). There is also enhanced variability in prenatal *MEF2C* expression compared to postnatal expression (*p* = 0.02)—an effect likely due to marked change from first to second and third trimesters of gestation. *MEF2C* expression increases substantially from first to second trimester of gestation and continues high levels of expression until around 2 years of age, when expression tapers off and becomes stable throughout the rest of the lifespan (Fig. 3d).

Finally, we tested whether *MEF2C* is dysregulated in induced pluripotent stem cells (iPSC) from male patients with autism and whether the directionality of such dysregulation is congruent with the directionality of DHT-influence on *MEF2C* expression. To achieve this aim, we re-examined a recent RNA-seq dataset, which examined iPSC, neural progenitor cells, and neurons grown from fibroblasts of male cases of autism [10]. An ANOVA examining all cell types identified a Diagnosis⁎Cell Type interaction (*F* = 6.89, *p* = 0.0047). Congruent with the directionality of DHT upregulation, *MEF2C* is also upregulated in iPSCs from male patients with autism (*t* = 3.68, *p* = 0.0035, *Cohen’s d* = 1.42). In contrast, no dysregulation is observed in neural progenitor cells (*t* = 0.12, *p* = 0.90, *Cohen’s d* = 0.11) or neurons in autism (*t* = 2.19, *p* = 0.052, *Cohen’s d* = 1.31) (Fig. 3e).

## Discussion

In this study, we have shown that variation in testosterone during midgestational prenatal development has long-lasting effects on how specific networks comprising the core of the social brain, the DMN, are organized. In line with hypotheses about sex-specific mechanisms, we identified that the fetal programming impact of testosterone on later circuit-level organization of the DMN depends on whether an individual is male or female. These findings are compatible with other normative large-scale work on sex differences in DMN connectivity. Two large independent studies on adults utilizing the 1000 Functional Connectomes or UK Biobank datasets now replicably show that default mode connectivity is subtly on-average stronger in females than in males [49, 50]. While these prior and much larger studies find these subtle DMN sex differences, it is noteworthy that sex differences in DMN connectivity were not apparent in the current dataset (*t* = 0.23, *p* = 0.81; Supplementary Fig. 5). This result is most likely due to lack of statistical power for identifying the subtle sex differences that prior and much larger studies have identified. Nevertheless, the directionality of documented on-average DMN sex differences in functional connectivity are congruent with the directionality of the male-specific influence shown here—FT reduces later DMN connectivity, and thus could be one explanation behind the on-average lower DMN connectivity in males observed in other studies [49, 50].

These findings are also particularly relevant for understanding how the prenatal environmental shapes early neurobiological mechanisms and heightens male-susceptibility for early-onset neurodevelopmental conditions. Autism is an early neurodevelopmental condition of particular relevance here, given the 3:1 sex ratio [12] alongside other work implicating elevated prenatal steroidogenic activity [7, 18]. The hallmark features of autism are profound difficulties with early social-communication and social behavior, which has led to many investigations on the possible link with default mode network organization and function. The two core DMN subsystems identified in this work are known for the importance in both mentalizing and self-referential cognition and these domains along with the DMN circuitry underlying them are known to be atypical in males with autism [51–54]. The current results suggest that FT could act as a male-specific mechanism that reduces connectivity between core default mode subsystems linked to domains that are highly important for social-communication.

Showing that FT influences later functional organization within the human brain is the first step in understanding the developmental biology behind this effect. Imperative in working towards this translational goal is the need to understand how FT might have male-specific biological effects on developing neural circuits for the social brain, such as the DMN. Using a human neural stem cell model, we identified a subset of genes that are differentially expressed after androgen treatment. In an important link to autism, this set of genes is enriched in a number of high-impact genes known to be causes of syndromic autism and intellectual disability. This finding underscores a prior paper [28] utilizing the same dataset that came across similar findings albeit with a different approach for differential expression analysis. However, this finding makes some specific inferences with regards to enrichments with the ‘‘syndromic’’ category of variants (*n* = 102) labeled within SFARI Gene, whereas the prior paper looked for enrichment with any of *n* = 235 genes annotated within SFARI Gene.

In keeping with the relevant early phase of neurodevelopment for hNSCs, the DHT manipulation affected many genes involved in important neurodevelopmental processes including neurogenesis, cell differentiation, pattern specification and regionalization, and morphogenesis. These findings are also in line with previous work [28] whereby independent experiments showed that DHT enhances cell proliferation and prevents cell death during neuronal differentiation under nutrient-deprived conditions. These processes are hypothesized to be some of the earliest key prenatal processes that are affected in autism and dysregulation of these processes can have multifinal outcomes and pleiotropic effects later in life including atypical circuit formation and function [55]. Androgen activity can be considered as a gene regulatory influence over prenatal brain development given that such sex hormones work via androgen receptor signaling to directly affect transcription of many other genes. This work further supports the idea that androgens can exert regulatory impact over prenatal neurobiological processes that are highly linked to male-biased conditions like autism.

Going beyond the list of DHT-dysregulated genes, we went further to pinpoint a subset of DHT-influenced genes that are also highly relevant specifically for the cortical midline DMN subsystem that shows a male-specific influence of FT. This subset of DHT-dysregulated and cortical midline DMN-relevant genes was enriched for a variety of synaptic processes and potentially highlights important biological mechanisms in prenatal development that androgens act on. For example, the top enrichment in such genes was for excitatory postsynaptic potentials and includes genes for glutamate receptors such as *GRIK2*, *GRIA1*, and *GRIA2*, all of which are upregulated by DHT. Also included in this enrichment is *MEF2C*, which is upregulated by DHT, and is known to alter excitation-inhibition (E-I) imbalance [56, 57]. Also relevant to E-I imbalance is *NPTX2*, which has effects on GluA4-containing AMPA receptors [58] and synaptogenesis [59]. DHT also upregulates sodium-gated ion channel genes (*SCN3A*, *SCN9A*), which have spatial similarity expression with cortical midline DMN and could also be relevant to E-I imbalance. All of these effects whereby DHT upregulates expression of such genes may point to the importance of E-I imbalance in androgen-impact on early prenatal brain development. This is particularly relevant to autism given common theoretical views about E-I imbalance in autism [60]. Because these genes are highly expressed in spatial patterns resembling the DMN network we find associated with FT in rsfMRI, it may be that these are key mechanisms of prenatal androgen-impact on early developing social brain DMN circuit formation and maintenance.

While there are many genes from this work that could be focused on, we particularly highlight the role of *MEF2C*. Ample evidence supports the involvement of *MEF2C* as a candidate mechanism involved in male-biased early developmental conditions such as autism and intellectual disability [4, 57, 61–64]. Developmentally, *MEF2C* is highly expressed during prenatal development (Fig. 3d). Within prenatal development *MEF2C* has the potential to be a transcriptional regulator of a number of autism-associated genes— *MEF2C* binding motifs are enriched in upstream regions of genes within prenatal gene co-expression modules that harbor a number of autism-associated genes [4]. Congruent with the idea that *MEF2C* is important for many prenatal neurodevelopmental processes, other work has shown it is involved in neurogenesis, cell differentiation, maturation, and migration [65–67] as well as having later roles to play in experience-dependent synaptic development and cell death [56, 57, 68, 69]. *MEF2C* is also a known downstream target of the androgen receptor (AR), thus making it highly susceptible to androgen-dependent transcriptional influence [45]. *MEF2C* is also one of the most important transcription factor genes influencing sex differences in human brain gene expression—*MEF2C* differentially targets genes as a function of sex, and the magnitude of this sex-differential targeting explains a large percentage of variance in sex differences in gene expression of its putative targets [48]. Our work further underscores *MEF2C*’s involvement in male-specific mechanisms behind atypical neurodevelopment, and particularly highlights how *MEF2C* could be one of many candidates behind fetal programming effects of androgens on developing social brain circuits such as the DMN. Compatible with effects on early neurogenesis and differentiation, we find evidence that *MEF2C* expression is dysregulated in the same direction as FT influence in iPS cells in male cases with autism. Thus, important future work could examine *AR* targeting of *MEF2C* and its effect on early neurogenesis and differentiation processes. Finally, while available data suggests that rare deleterious *MEF2C* mutations are not more prevalent in males vs. females with autism or intellectual disability [70–72], it is unclear whether sex effects would exist in more subtle missense mutations or whether effects might appear with much larger sample size. It will be important in future work to examine these questions with larger datasets.

There are some important caveats and limitations to be discussed. As illustrated in Fig. 1c, it is noteworthy that FT exhibits a much larger range of variability in males than females. From the current data we cannot determine whether females might show a similar effect if FT variability was enhanced to a similar range as that of males. Thus, the interpretations must be tempered by the caveat that the current data cannot tell us whether the observed effects are due to a fundamental difference in the how males vs. females respond to FT or whether the effect is simply a matter of difference in how much FT males and females are exposed to. Further work examining females with a larger range of FT levels is needed to better understand the sex-specific effects of FT exposure on later development. Second, the current work on hNSCs primarily shows how testosterone may have important actions on early neural development via conversion to DHT and action on the androgen receptor. However, future work could examine other routes of influence, such as aromatization of testosterone to estradiol and actions through the estrogen receptor. Furthermore, prior work has shown that the gene *RORA* is influenced by androgens and estrogens and can have further regulatory influence over aromatase levels and act to reduce estrogen levels [73]. *RORA* and aromatase levels are both reduced in frontal cortex in autism and are highly related to each other [73, 74]. This work suggests that routes of influence via the AR may be more likely implicated in autism. Nonetheless, future work should investigate the role of testosterone conversion to estradiol and actions via the estrogen receptor and how such mechanisms interact with *RORA* in autism.

In conclusion, we find that variation in FT during midgestational periods of prenatal development has a sex-specific impact on later human brain development. In particular, FT reduces functional connectivity in adolescence between social brain DMN subsystems in males, but has no effect on DMN functional connectivity in females. This sex-specific influence in early prenatal development was modeled in hNSCs to discover how androgens may act as a transcriptional influence on genes that are highly relevant in the adult brain for specific DMN-related circuitry. Here, we discovered that DHT-dysregulated genes are enriched for syndromic causes of autism and intellectual disability and are highly enriched for genes that are spatially expressed in a similar pattern to the cortical midline DMN subsystem. These DHT-dysregulated and DMN-relevant genes (e.g., *MEF2C*) are involved in a variety of synaptic processes and may affect excitation-inhibition balance. These affected processes are congruent with the idea that FT may exert fetal programming influence on genes that play roles in biological processes that are integral for later circuit formation and maintenance. DHT also plays a prominent role in dysregulating genes involved in many early neurodevelopmental processes such as neurogenesis, cell differentiation, patterning, and regionalization. This effect is compatible with the idea that androgens can affect genes that may have pleiotropic roles at early and later phases of brain development that link early cell proliferation and differentiation to later synaptic organization. *MEF2C* is one particular gene with such early and late developmental roles. *MEF2C* is also highly relevant to cortical midline DMN circuitry and is highly associated with male-biased conditions such as autism and intellectual disability. *MEF2C* is upregulated in hNSCs by DHT. In an important link with autism, we find similar *MEF2C* upregulation in iPS cells, but not neural progenitor cells or neurons from male cases of autism. This work highlights that prenatal androgens may have male-specific influence over early prenatal neurodevelopmental processes that can potentially manifest as long-lasting influence over social brain circuitry. These effects may help explain normative sex differences in brain and behavior as well as increased male-susceptibility to conditions such as autism.

## Electronic supplementary material

Supplementary Results and Figures

## References

[1] Barker DJ (1998). In utero programming of chronic disease. Clin Sci (Lond).

[2] Gluckman PD, Hanson MA, Cooper C, Thornburg KL (2008). Effect of in utero and early-life conditions on adult health and disease. N Engl J Med.

[3] Seckl JR, Holmes MC (2007). Mechanisms of disease: glucocorticoids, their placental metabolism and fetal ‘programming’ of adult pathophysiology. Nat Clin Pract Endocrinol Metab.

[4] Parikshak NN, Luo R, Zhang A, Won H, Lowe JK, Chandran V (2013). Integrative functional genomic analyses implicate specific molecular pathways and circuits in autism. Cell.

[5] Willsey AJ, Sanders SJ, Li M, Dong S, Tebbenkamp AT, Muhle RA (2013). Coexpression networks implicate human midfetal deep cortical projection neurons in the pathogenesis of autism. Cell.

[6] Lombardo MV, Moon HM, Su J, Palmer TD, Courchesne E, Pramparo T (2018). Maternal immune activation dysregulation of the fetal brain transcriptome and relevance to the pathophysiology of autism spectrum disorder. Mol Psychiatry.

[7] Baron-Cohen S, Auyeung B, Norgaard-Pedersen B, Hougaard DM, Abdallah MW, Melgaard L (2015). Elevated fetal steroidogenic activity in autism. Mol Psychiatry.

[8] Stoner R, Chow ML, Boyle MP, Sunkin SM, Mouton PR, Roy S (2014). Patches of disorganization in the neocortex of children with autism. N Engl J Med.

[9] Courchesne E, Mouton PR, Calhoun ME, Semendeferi K, Ahrens-Barbeau C, Hallet MJ (2011). Neuron number and size in prefrontal cortex of children with autism. JAMA: J Am Med Assoc.

[10] Marchetto MC, Belinson H, Tian Y, Freitas BC, Fu C, Vadodaria K (2017). Altered proliferation and networks in neural cells derived from idiopathic autistic individuals. Mol Psychiatry.

[11] Rutter M, Caspi A, Moffitt TE (2003). Using sex differences in psychopathology to study causal mechanisms: unifying issues and research strategies. J Child Psychol Psychiatry.

[12] Loomes R, Hull L, Mandy WPL (2017). What is the male-to-female ratio in autism spectrum disorder? A systematic review and meta-analysis. J Am Acad Child Adolesc Psychiatry.

[13] Lai MC, Lombardo MV, Auyeung B, Chakrabarti B, Baron-Cohen S (2015). Sex/gender differences and autism: setting the scene for future research. J Am Acad Child Adolesc Psychiatry.

[14] Hull L, Petrides KV, Allison C, Smith P, Baron-Cohen S, Lai MC (2017). “Putting on my best normal”: social camouflaging in adults with autism spectrum conditions. J Autism Dev Disord.

[15] Robinson EB, Lichtenstein P, Anckarsater H, Happe F, Ronald A (2013). Examining and interpreting the female protective effect against autistic behavior. Proc Natl Acad Sci USA.

[16] Jacquemont S, Coe BP, Hersch M, Duyzend MH, Krumm N, Bergmann S (2014). A higher mutational burden in females supports a “female protective model” in neurodevelopmental disorders. Am J Hum Genet.

[17] Baron-Cohen S (2002). The extreme male brain theory of autism. Trends Cogn Sci.

[18] Kosidou K, Dalman C, Widman L, Arver S, Lee BK, Magnusson C (2016). Maternal polycystic ovary syndrome and the risk of autism spectrum disorders in the offspring: a population-based nationwide study in Sweden. Mol Psychiatry.

[19] Kosidou K, Dalman C, Widman L, Arver S, Lee BK, Magnusson C (2017). Maternal polycystic ovary syndrome and risk for attention-deficit/hyperactivity disorder in the offspring. Biol Psychiatry.

[20] Cherkosv A, Pohl A, Allison C, Zhang H, Payne RA, Baron-Cohen S. Polycystic ovary syndrome and autism: A test of the prenatal sex steroid theory. Transl Psychiatry 2018. 10.1038/s41398-018-0186-7 PMC6068102 30065244

[21] Auyeung B, Baron-Cohen S, Ashwin E, Knickmeyer R, Taylor K, Hackett G (2009). Fetal testosterone and autistic traits. Br J Psychol.

[22] Auyeung B, Taylor K, Hackett G, Baron-Cohen S (2010). Foetal testosterone and autistic traits in 18 to 24-month-old children. Mol Autism.

[23] Auyeung B, Lombardo MV, Baron-Cohen S (2013). Prenatal and postnatal hormone effects on the human brain and cognition. Pflug Arch: Eur J Physiol.

[24] Bos PA, Panksepp J, Bluthe RM, van Honk J (2012). Acute effects of steroid hormones and neuropeptides on human social-emotional behavior: a review of single administration studies. Front Neuroendocrinol.

[25] Whitehouse AJ, Mattes E, Maybery MT, Sawyer MG, Jacoby P, Keelan JA (2012). Sex-specific associations between umbilical cord blood testosterone levels and language delay in early childhood. J Child Psychol Psychiatry.

[26] Hines M (2004). Brain gender.

[27] Smail PJ, Reyes FI, Winter JSD, Fairman C. The fetal hormonal environment and its effect on the morphogenesis of the genital system. In: Kogan SJ, Hafez ESE, editors. Pediatric andrology. Martinus Nijhoff: The Hague; 1981. p 9–19.

[28] Quartier A, Chatrousse L, Redin C, Keime C, Haumesser N, Maglott-Roth A, et al. Genes and pathways regulated by androgens in human neural cells, potential candidates for the male excess in autism spectrum disorders. Biol Psychiatry. 2018. 10.1016/j.biopsych.2018.01.002. 10.1016/j.biopsych.2018.01.002 29428674

[29] Kundu P, Brenowitz ND, Voon V, Worbe Y, Vertes PE, Inati SJ (2013). Integrated strategy for improving functional connectivity mapping using multiecho fMRI. Proc Natl Acad Sci USA.

[30] Kundu P, Inati SJ, Evans JW, Luh WM, Bandettini PA (2012). Differentiating BOLD and non-BOLD signals in fMRI time series using multi-echo EPI. Neuroimage.

[31] Lombardo MV, Auyeung B, Holt RJ, Waldman J, Ruigrok ANV, Mooney N (2016). Improving effect size estimation and statistical power with multi-echo fMRI and its impact on understanding the neural systems supporting mentalizing. Neuroimage.

[32] Smith SM, Vidaurre D, Beckmann CF, Glasser MF, Jenkinson M, Miller KL (2013). Functional connectomics from resting-state fMRI. Trends Cogn Sci.

[33] Marrelec G, Krainik A, Duffau H, Pelegrini-Issac M, Lehericy S, Doyon J (2006). Partial correlation for functional brain interactivity investigation in functional MRI. Neuroimage.

[34] Smith SM, Miller KL, Salimi-Khorshidi G, Webster M, Beckmann CF, Nichols TE (2011). Network modelling methods for FMRI. Neuroimage.

[35] Wager TD, Keller MC, Lacey SC, Jonides J (2005). Increased sensitivity in neuroimaging analyses using robust regression. Neuroimage.

[36] Boissart C, Poulet A, Georges P, Darville H, Julita E, Delorme R (2013). Differentiation from human pluripotent stem cells of cortical neurons of the superficial layers amenable to psychiatric disease modeling and high-throughput drug screening. Transl Psychiatry.

[37] Robinson MD, Oshlack A (2010). A scaling normalization method for differential expression analysis of RNA-seq data. Genome Biol.

[38] Love MI, Huber W, Anders S (2014). Moderated estimation of fold change and dispersion for RNA-seq data with DESeq2. Genome Biol.

[39] Law CW, Chen Y, Shi W, Smyth GK (2014). voom: Precision weights unlock linear model analysis tools for RNA-seq read counts. Genome Biol.

[40] Storey JD (2002). A direct approach to false discovery rates. J R Stat Soc, Ser B.

[41] Gorgolewski KJ, Fox AS, Chang L, Schafer A, Arelin K, Burmann I (2014). Tight fitting genes: Finding relations between statistical maps and gene expression patterns. F1000 Posters.

[42] Hawrylycz M, Miller JA, Menon V, Feng D, Dolbeare T, Guillozet-Bongaarts AL (2015). Canonical genetic signatures of the adult human brain. Nat Neurosci.

[43] Hawrylycz MJ, Lein ES, Guillozet-Bongaarts AL, Shen EH, Ng L, Miller JA (2012). An anatomically comprehensive atlas of the adult human brain transcriptome. Nature.

[44] Verhagen J, Wagenmakers EJ (2014). Bayesian tests to quantify the result of a replication attempt. J Exp Psychol Gen.

[45] Wyce A, Bai Y, Nagpal S, Thompson CC (2010). Research resource: The androgen receptor modulates expression of genes with critical roles in muscle development and function. Mol Endocrinol.

[46] Werling DM, Parikshak NN, Geschwind DH (2016). Gene expression in human brain implicates sexually dimorphic pathways in autism spectrum disorders. Nat Commun.

[47] Gershoni M, Pietrokovski S (2017). The landscape of sex-differential transcriptome and its consequent selection in human adults. BMC Biol.

[48] Chen C-Y, Lopes-Ramos CM, Kuijjer ML, Paulson JN, Sonawane AR, Fagny M, et al. Sexual dimorphism in gene expression and regulatory networks across human tissues. *bioRxiv* 2016. 10.1101/082289

[49] Biswal BB, Mennes M, Zuo XN, Gohel S, Kelly C, Smith SM (2010). Toward discovery science of human brain function. Proc Natl Acad Sci USA.

[50] Ritchie SJ, Cox SR, Shen X, Lombardo MV, Reus LM, Alloza C, et al. Sex differences in the adult human brain: Evidence from 5216 UK biobank participants. Cereb Cortex. 2018. 2959–2975. 10.1093/cercor/bhy109 PMC6041980 29771288

[51] Buckner RL, Carroll DC (2006). Self-projection and the brain. Trends Cogn Sci.

[52] Lombardo MV, Chakrabarti B, Bullmore ET, Sadek SA, Wheelwright SJ, Pasco G (2010). Atypical neural self-representation in autism. Brain.

[53] Lombardo MV, Chakrabarti B, Bullmore ET, Wheelwright SJ, Sadek SA, Suckling J (2010). Shared neural circuits for mentalizing about the self and others. J Cogn Neurosci.

[54] Padmanabhan A, Lynch CJ, Schaer M, Menon V (2017). The Default Mode Network in Autism. Biol Psychiatry Cogn Neurosci Neuroimaging.

[55] Courchesne E, Pramparo T, Gazestani VH, Lombardo MV, Pierce K, Lewis N. The ASD living biology: From cell proliferation to clinical phenotype. Mol. Psychiatry. 2018. 10.1038/s41380-018-0056-y 10.1038/s41380-018-0056-y PMC6309606 29934544

[56] Harrington AJ, Raissi A, Rajkovich K, Berto S, Kumar J, Molinaro G, et al. MEF2C regulates cortical inhibitory and excitatory synapses and behaviors relevant to neurodevelopmental disorders. Elife. 2016; 5. 10.7554/eLife.20059 PMC5094851 27779093

[57] Tu S, Akhtar MW, Escorihuela RM, Amador-Arjona A, Swarup V, Parker J (2017). NitroSynapsin therapy for a mouse MEF2C haploinsufficiency model of human autism. Nat Commun.

[58] Pelkey KA, Barksdale E, Craig MT, Yuan X, Sukumaran M, Vargish GA (2015). Pentraxins coordinate excitatory synapse maturation and circuit integration of parvalbumin interneurons. Neuron.

[59] Appelbaum L, Wang G, Yokogawa T, Skariah GM, Smith SJ, Mourrain P (2010). Circadian and homeostatic regulation of structural synaptic plasticity in hypocretin neurons. Neuron.

[60] Rubenstein JL, Merzenich MM (2003). Model of autism: increased ratio of excitation/inhibition in key neural systems. Genes Brain Behav.

[61] Neale BM, Kou Y, Liu L, Ma’ayan A, Samocha KE, Sabo A (2012). Patterns and rates of exonic de novo mutations in autism spectrum disorders. Nature.

[62] RKC Yuen, Merico D, Bookman M, LH J, Thiruvahindrapuram B, Patel RV (2017). Whole genome sequencing resource identifies 18 new candidate genes for autism spectrum disorder. Nat Neurosci.

[63] Lipton SA, Li H, Zaremba JD, McKercher SR, Cui J, Kang YJ (2009). Autistic phenotype from MEF2C knockout cells. Science.

[64] Redin C, Brand H, Collins RL, Kammin T, Mitchell E, Hodge JC (2017). The genomic landscape of balanced cytogenetic abnormalities associated with human congenital anomalies. Nat Genet.

[65] Li H, Radford JC, Ragusa MJ, Shea KL, McKercher SR, Zaremba JD (2008). Transcription factor MEF2C influences neural stem/progenitor cell differentiation and maturation in vivo. Proc Natl Acad Sci USA.

[66] Li Z, McKercher SR, Cui J, Nie Z, Soussou W, Roberts AJ (2008). Myocyte enhancer factor 2C as a neurogenic and antiapoptotic transcription factor in murine embryonic stem cells. J Neurosci.

[67] Novara F, Beri S, Giorda R, Ortibus E, Nageshappa S, Darra F (2010). Refining the phenotype associated with MEF2C haploinsufficiency. Clin Genet.

[68] Elmer BM, Estes ML, Barrow SL, McAllister AK (2013). MHCI requires MEF2 transcription factors to negatively regulate synapse density during development and in disease. J Neurosci.

[69] Rajkovich KE, Loerwald KW, Hale CF, Hess CT, Gibson JR, Huber KM (2017). Experience-dependent and differential regulation of local and long-range excitatory neocortical circuits by postsynaptic Mef2c. Neuron.

[70] Deciphering Developmental Disorders S. (2017). Prevalence and architecture of de novo mutations in developmental disorders. Nature.

[71] Sanders SJ, He X, Willsey AJ, Ercan-Sencicek AG, Samocha KE, Cicek AE (2015). Insights into autism spectrum disorder genomic architecture and biology from 71 risk loci. Neuron.

[72] Novara F, Rizzo A, Bedini G, Girgenti V, Esposito S, Pantaleoni C (2013). MEF2C deletions and mutations versus duplications: a clinical comparison. Eur J Med Genet.

[73] Sarachana T, Xu M, Wu RC, Hu VW (2011). Sex hormones in autism: androgens and estrogens differentially and reciprocally regulate RORA, a novel candidate gene for autism. PLoS ONE.

[74] Nguyen A, Rauch TA, Pfeifer GP, Hu VW (2010). Global methylation profiling of lymphoblastoid cell lines reveals epigenetic contributions to autism spectrum disorders and a novel autism candidate gene, RORA, whose protein product is reduced in autistic brain. FASEB J.

