# Supplementary material for: Sex-specific impact of prenatal androgens on social brain default mode subsystems
Source: Mol Psychiatry. 2018 Aug 13;25(9):2175–88. doi: 10.1038/s41380-018-0198-y (PMC7473837; doi:10.1038/s41380-018-0198-y)
Supplement: Supplementary file 1 — Supplementary Results and Figures [file 41380_2018_198_MOESM1_ESM.pdf]

Supplementary Results and Figures

Supplementary Figure 1: *Association between FT and DMN connectivity ignoring sex.*

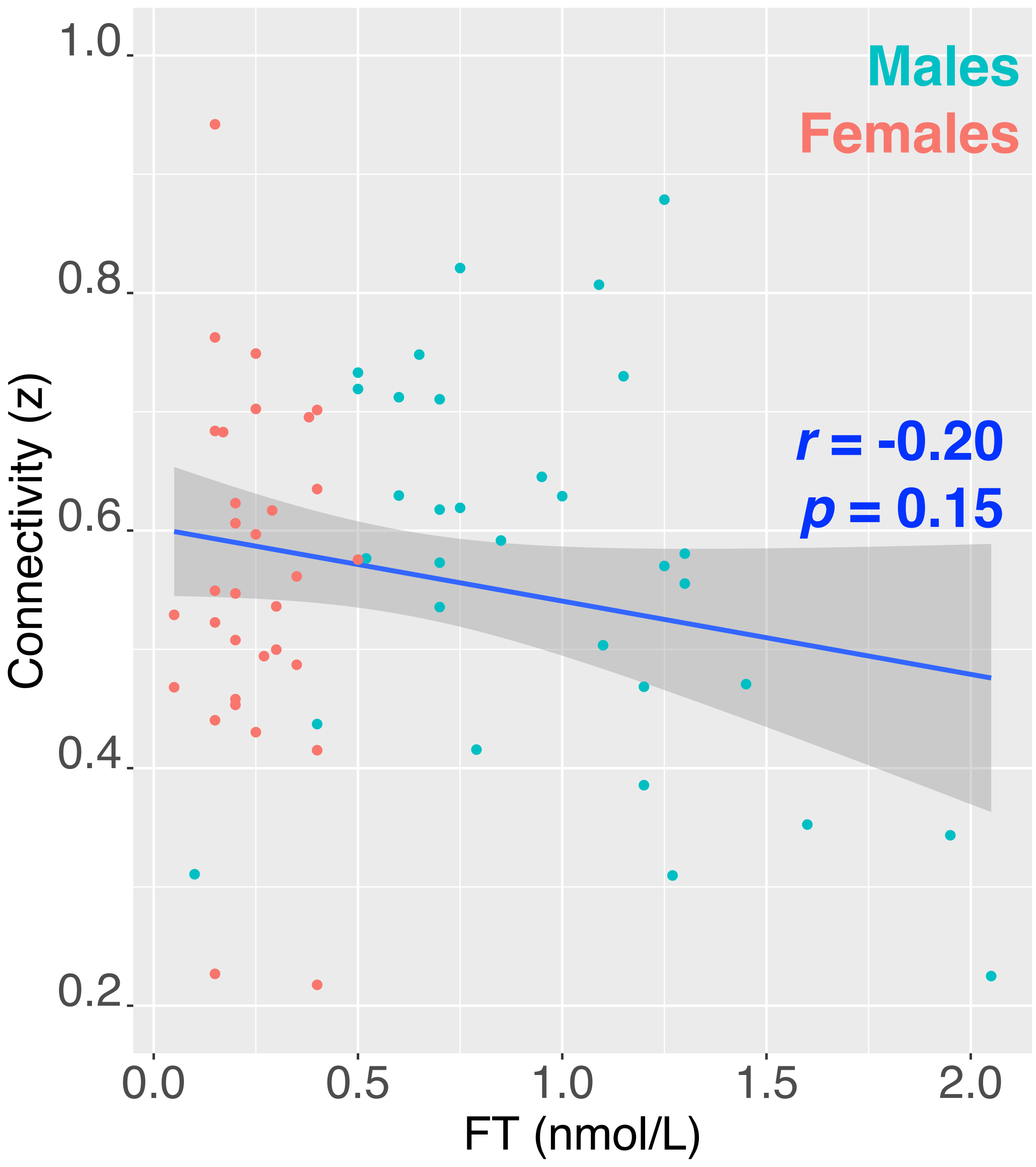

Supplementary Figure 2: *Relationship between FT and current testosterone level in adolescence.*

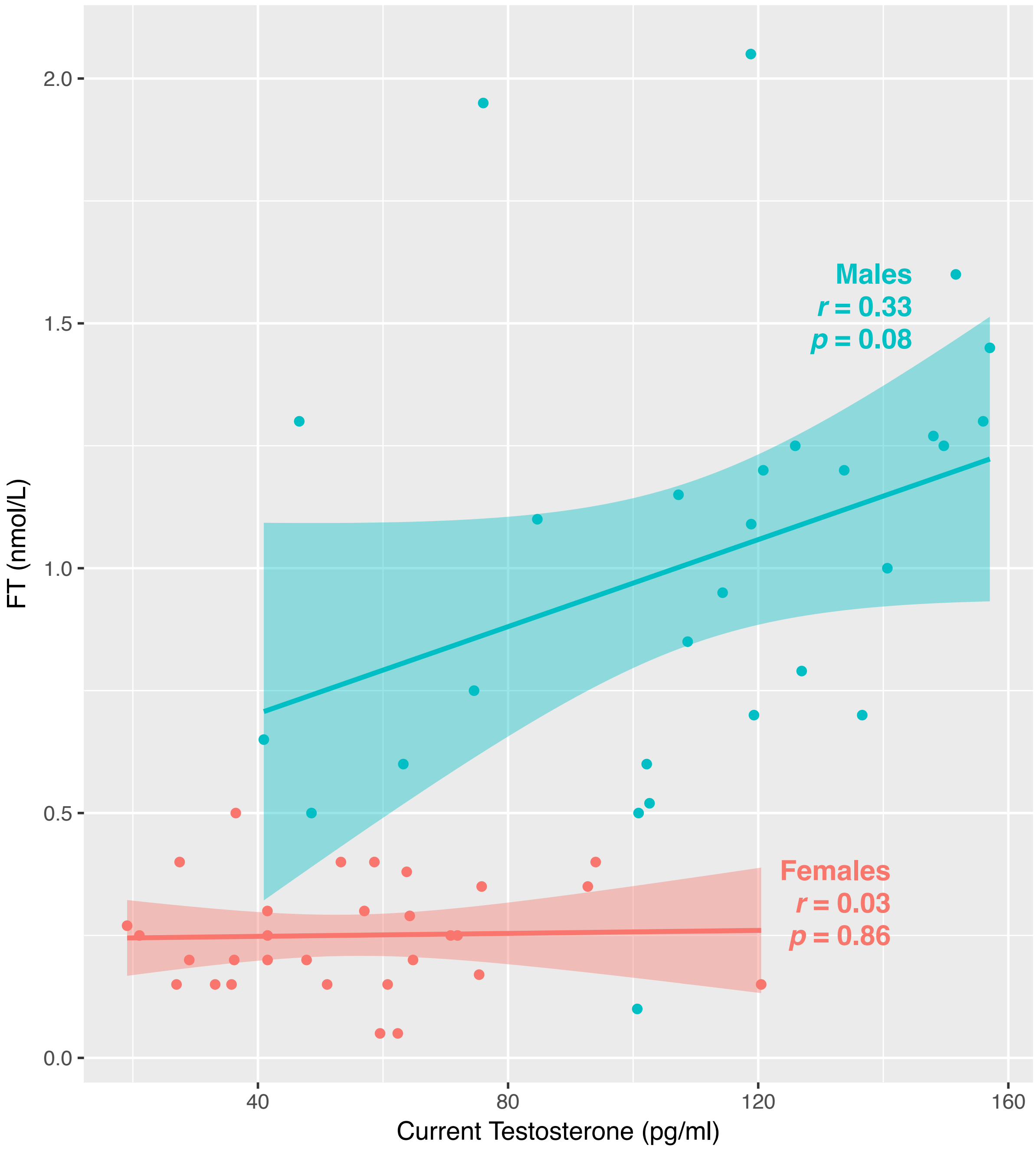

Supplementary Figure 3: *Relationship between current testosterone and DMN connectivity*

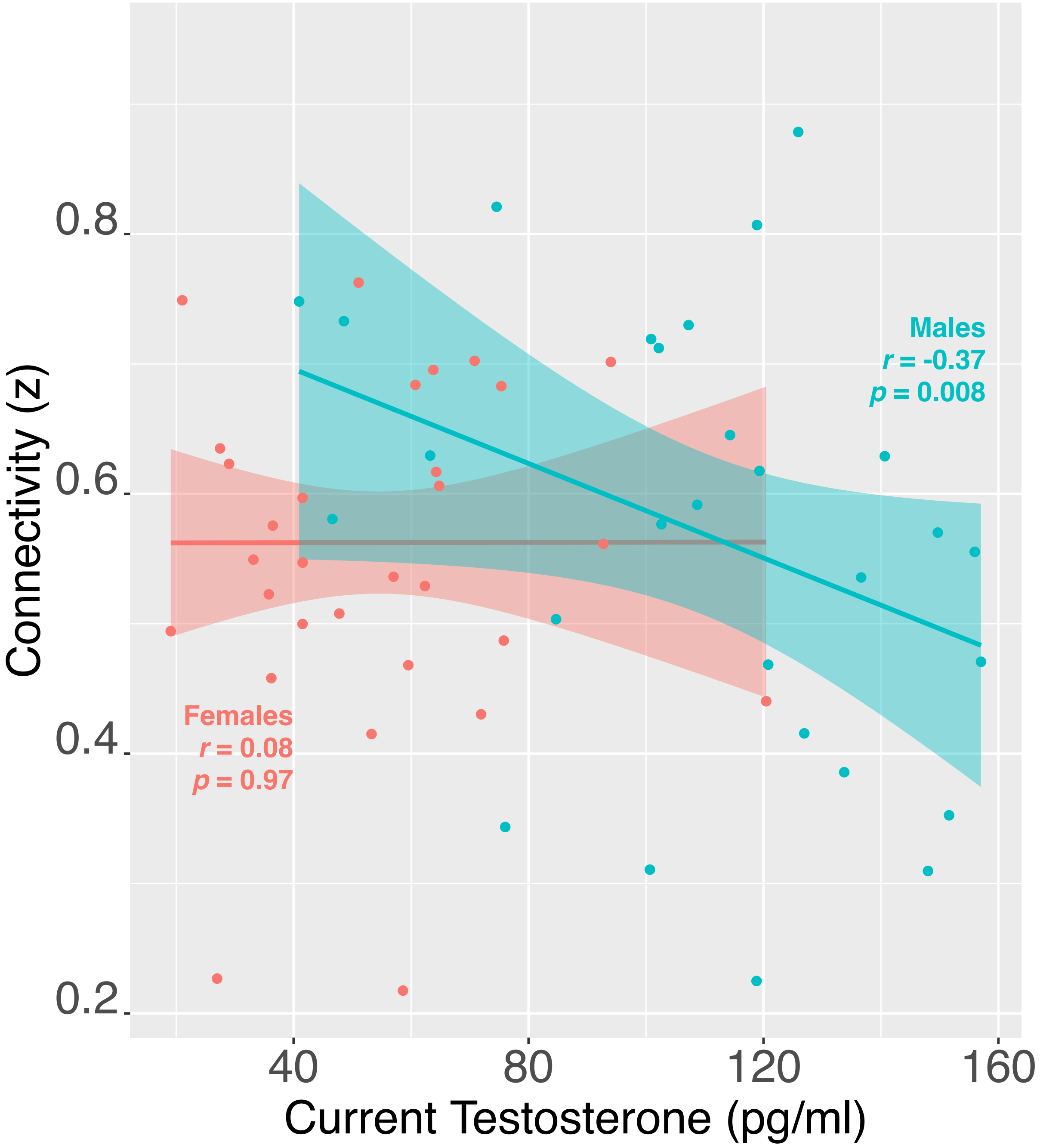

Supplementary Figure 4: *Biological process enrichment of DHT-upregulated (A) and downregulated (B) genes sets and plots of specific autism-associated genes (C).*

Quartier and colleagues recently reported DHT dysregulates genes were enriched for autism-associated genes[1]. The DE analysis in this paper utilizes the same RNA-seq dataset, but performs the DE analysis differently than that of Quartier and colleagues. Therefore, we re-examined this question with the current study's DE lists. Upon examining SFARI Gene Scoring categories for overlap with autism-associated genes, we find that a number of genes in the 'Syndromic' category are enriched in genes that are influenced by DHT manipulation (OR = 2.16, p = 0.02). These genes include *AHI1*, *ASXL3*, *CHD2*, *DHCR7*, *HCN1*, *MEF2C*, *PAX6*, *PRODH*, *PTEN*, and *SCN1A*. *ASXL3* and *PTEN* are also classified in the high-confidence category. No other gene scoring categories were enriched. However, there were many other notable autism-associated genes influenced by DHT, including *NLGN4X*, *NRXN3*, *FOXP1*, and *SCN9A* (Supplementary Figure 4C). Thus, this analysis highlights a largely-overlapping finding with the prior paper by Quartier et al., [1] showing that many syndromic causes of autism are enriched within the DHT-dysregulated gene set.

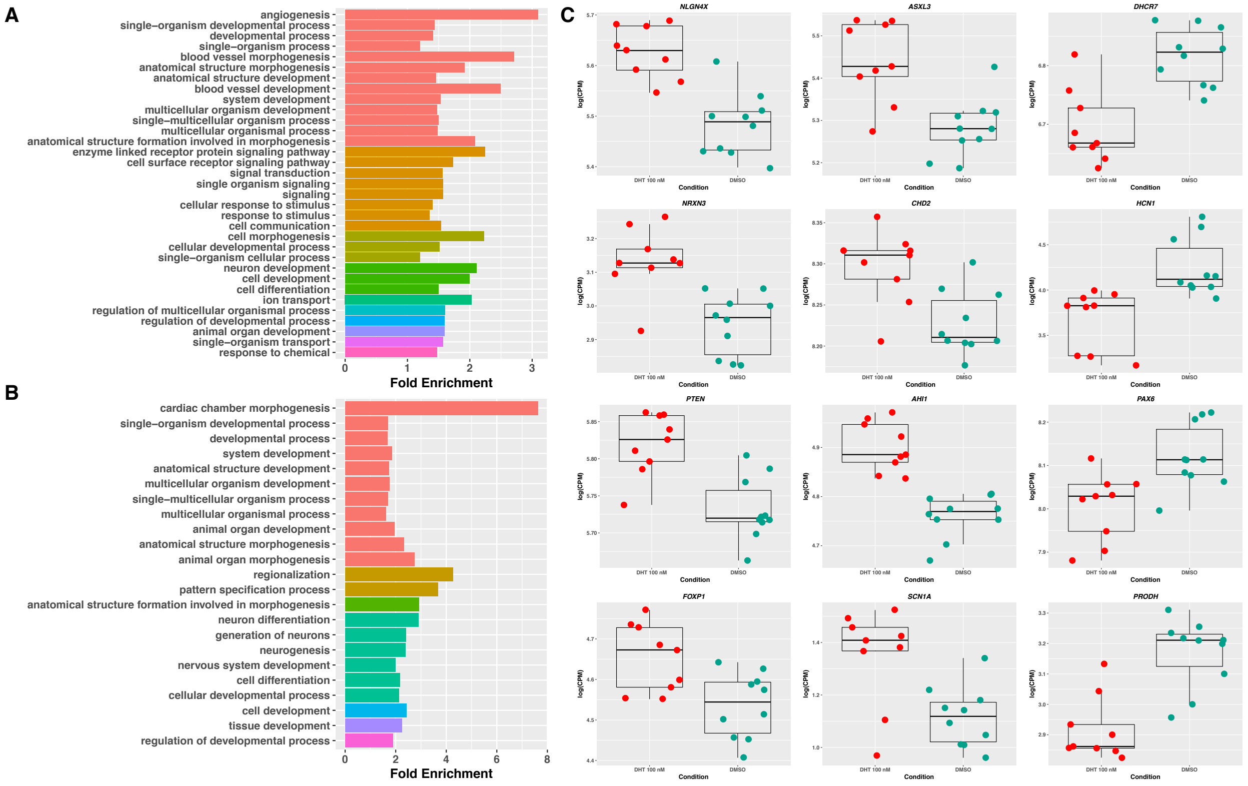

Supplementary Figure 5: *DMN connectivity across males and females*

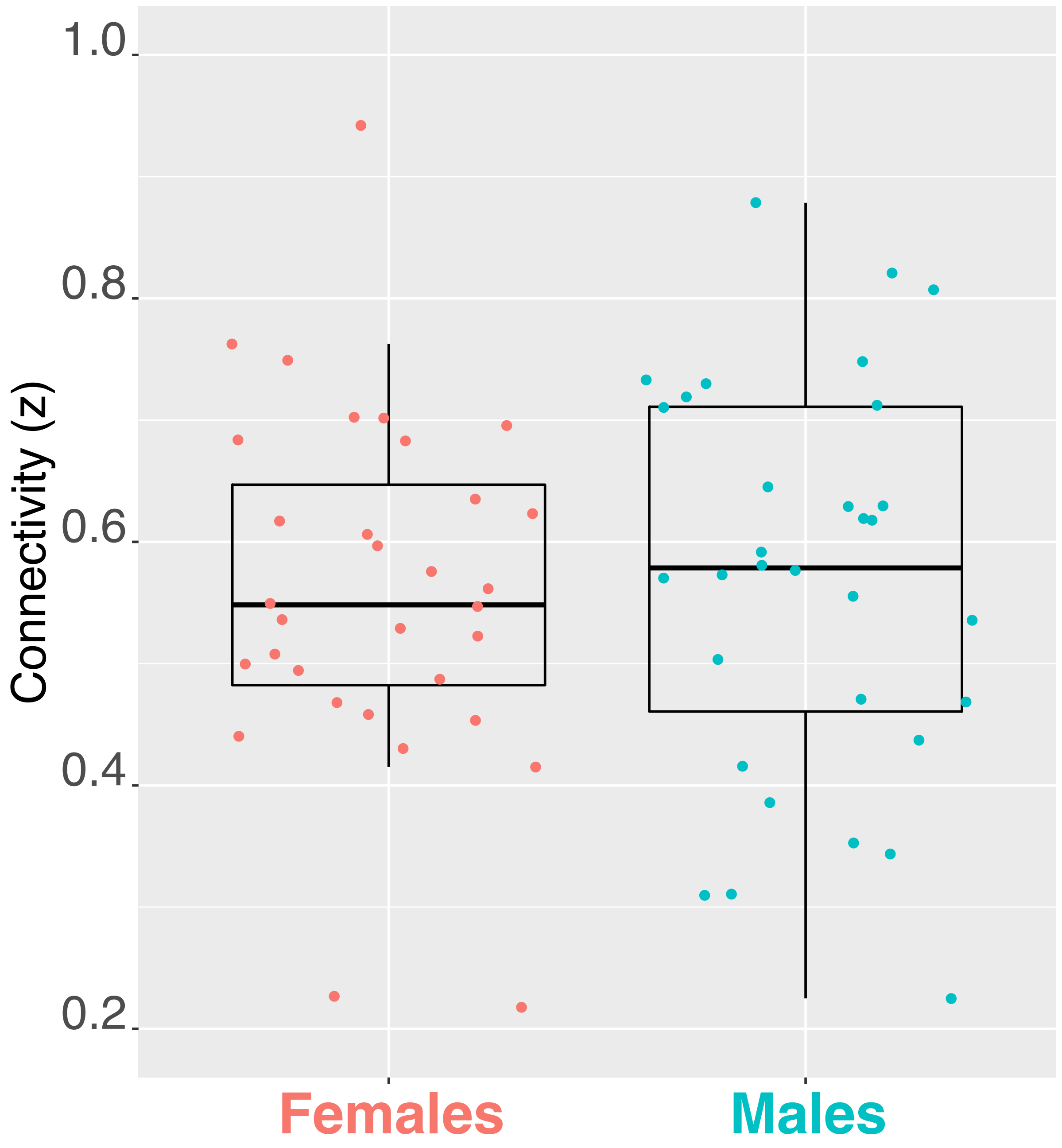

References

1. Quartier A, Chatrousse L, Redin C, Keime C, Haumesser N, Maglott-Roth A *et al.* Genes and pathways regulated by androgens in human neural cells, potential candidates for the male excess in autism spectrum disorders. *Biol Psychiatry* 2018.
